# Multi-locus genomic signatures of local adaptation to snow across the landscape in California populations of a willow leaf beetle

**DOI:** 10.1098/rspb.2023.0630

**Published:** 2023-08-30

**Authors:** Abigail G. Keller, Elizabeth P. Dahlhoff, Ryan Bracewell, Kamalakar Chatla, Doris Bachtrog, Nathan E. Rank, Caroline M. Williams

**Affiliations:** ^1^ Department of Integrative Biology, University of California, Berkeley, CA, USA; ^2^ Department of Biology, Santa Clara University, Santa Clara, CA, USA; ^3^ Department of Biology, Indiana University Bloomington, Bloomington, IN, USA; ^4^ Department of Biology, Sonoma State University, Rohnert Park, CA, USA

**Keywords:** climate change, cold tolerance, insect, local adaptation, landscape genomics, winter

## Abstract

Organisms living in mountains contend with extreme climatic conditions, including short growing seasons and long winters with extensive snow cover. Anthropogenic climate change is driving unprecedented, rapid warming of montane regions across the globe, resulting in reduced winter snowpack. Loss of snow as a thermal buffer may have serious consequences for animals overwintering in soil, yet little is known about how variability in snowpack acts as a selective agent in montane ecosystems. Here, we examine genomic variation in California populations of the leaf beetle *Chrysomela aeneicollis*, an emerging natural model system for understanding how organisms respond to climate change. We used a genotype–environment association approach to identify genomic signatures of local adaptation to microclimate in populations from three montane regions with variable snowpack and a coastal region with no snow. We found that both winter-associated environmental variation and geographical distance contribute to overall genomic variation across the landscape. We identified non-synonymous variation in novel candidate loci associated with cytoskeletal function, ion transport and membrane stability, cellular processes associated with cold tolerance in other insects. These findings provide intriguing evidence that variation in snowpack imposes selective gradients in montane ecosystems.

## 1. Introduction

Seasonality serves as one of the strongest and most ubiquitous sources of environmental variation impacting natural systems, with distinct selective forces operating between periods of summer growth and reproduction and overwintering survival [1,2]. For small montane ectotherms, elevated and variable air temperatures during summertime can cause physiological stress during critical periods of reproduction, growth and development [3–5]. As hotter, drier summers become more common, upslope shifts in montane insect species are becoming more frequent, posing novel challenges at the limits of physiological tolerance [6,7]. For organisms that overwinter beneath the soil, snow cover is a key environmental factor influencing physiology and survival because snow buffers microclimate variability [8,9]. Climate change is causing more prevalent, intense and lengthy droughts, which in turn leads to more winters with a higher elevation snowline and lower total snowpack [10,11]. Reductions in snowpack may expose organisms overwintering in the soil to temperature extremes that cause physiological stress, reducing their overwintering survival and reproductive success at subsequent summer emergence. Recent declines in insect populations in montane environments documented across the globe demonstrate the urgency in gaining a clear understanding of how organisms cope with greater seasonal variability in temperature and precipitation in montane ecosystems [12–14]. Seasonal fluctuation can maintain genetic polymorphisms within populations [15,16], and variation in the extent and magnitude of seasonal fluctuations can generate spatial clines in allelic variants [17–19]. Elucidating how past climatic conditions have structured genetic variation and corresponding physiological responses for organisms in these habitats will be critical for predicting their responses to future environmental change.

Local adaptation, which occurs when resident genotypes have a higher relative fitness in their local habitat than genotypes originating from other habitats, is an important mechanism by which genetic variation is maintained in heterogeneous environments [20–22]. The extent and persistence of local adaptation are determined by a balance between natural selection for alleles that confer improved reproductive success in a particular microclimate and the homogenizing effects of gene flow and other neutral processes [22–26]. Neutral processes that influence patterns of genetic variation among populations include dispersal rates, colonization history, and population expansion and contraction, which in turn affect levels of genetic drift [24,25]. Local adaptation may be detected by identifying a stronger genetic variant ‘signal' from weaker, non-selective ‘noise' [27,28]. Unfortunately, selective climatic gradients, geography and migration corridors tend to covary, which complicates quantifying the relative contribution of selective and neutral evolutionary forces; thus, effects of isolation by distance (IBD) and population structure must be taken into account before patterns of genomic variation can be associated with selective features of the environment [29–32].

In this study, we investigated relationships between microclimatic factors and genetic variation in the willow leaf beetle *Chrysomela aeneicollis*, a well-described model species for understanding how climate change impacts montane ecosystems [18,33,34]. This insect is ideal for investigating processes of local adaptation in a region of high topographic and seasonal landscape heterogeneity [35]. During the brief summer growing season, this univoltine beetle species mates, lays eggs and undergoes one generation of larval development before new adults emerge and feed before winter returns [36]. They overwinter in the soil as freeze-tolerant adults for eight to nine months before emergence of reproductively mature adults [37,38].

In western North America, *C. aeneicollis* is found living on willows in cool, moist habitats separated by regions of arid or Mediterranean climates, resulting in highly fragmented distribution with little connectivity among populations [39,40]. In California, this species inhabits regions with distinct microclimate and seasonal characteristics: along high-elevation (2700–3400 m) streams and bogs in the Sierra Nevada (SN), in isolated montane populations on the edge of the Great Basin, and in low-elevation riparian habitats along the northern California coast. Within the SN, populations experience stressfully warm and cold temperatures throughout the year and their distribution is affected by seasonality and elevation, with populations contracting upslope and declining in abundance during droughts and growing in size and expanding to lower elevations after wet, snowy winters [4,33,36,38,41,42]. Despite these fluctuations in population size, SN populations have maintained high levels of heterozygosity at protein coding genes and other loci and show no deviation from Hardy–Weinberg expectations with respect to expected versus observed genotype frequencies [42,43], suggesting that they are sufficiently large to avoid bottlenecks and effects of inbreeding. Montane populations show evidence of substantial, stable genetic differentiation along a 60 km latitudinal gradient, from the South Fork of the Kings River in the south to Rock Creek in the north, with especially high divergence at mitochondrial loci and the metabolic enzyme locus *phosphoglucose isomerase*, *Pgi* [3,18,42,43]. Prior laboratory and field studies have also shown that effects of temperature on performance and fitness components vary among individuals with different nuclear and mitochondrial variants [33,35,42,44,45]. While extensive studies support the hypothesis that variation at metabolic loci such as *Pgi* and the mitochondrion reflect local adaptation [3,18,44,46], we lack information about how variation throughout the genome reflects the complex interaction of neutral and adaptive processes across the beetle's range.

Here, we address this gap by evaluating relationships between genomic variation and environmental conditions in locations where willow beetle populations occur in four distinct ecoregions of California [47]. We quantified differentiation at nuclear loci among populations in three montane regions in eastern California and populations in an isolated coastal area; this sampling design covers all known regions within California where this species is currently known to occur [39,40]. We identified selective microclimatic gradients that contribute to spatial patterns of potentially adaptive genomic variation across the landscape, then used this information to predict functions of newly identified genes that vary along microclimatic gradients to examine how genomic differentiation among these regions may contribute to local adaptation.

## 2. Results

### (a) Sequencing and marker filtering

Illumina sequencing generated 5.06 billion paired-end reads from 175 individuals in 12 populations (table 1), of which 4.05 billion total reads (80.1%) passed initial quality filters (per sample: mean = 23.1 million, s.d. = 7.9 million). The joint genotype calling workflow identified 12 million hard-filtered biallelic single nucleotide polymorphisms (SNPs) (electronic supplementary material, tables S1 and S2). We then used a conservative SNP filtering approach based on minor allele frequency (MAF), heterozygosity and inbreeding coefficient, resulting in 22 323 SNPs across all individuals and 12 populations. These SNPs were distributed evenly across the nuclear genome (electronic supplementary material, table S1). Filtering thresholds that contributed substantially to the reduced set of analysed SNPs were those that removed SNPs with a MAF < 0.01 (electronic supplementary material, table S2*b*) and that removed loci with low quality reads within populations (electronic supplementary material, table S2*c*).

**Table 1.** Localities and sample sizes for population genomic studies.

*ecoregion* population name	latitude	longitude	elevation (m)	*N* sites	*N* beetles (total)	year(s)^a^
*Sierra Nevada*						
Tuttle Creek (TC)	36.53779	−118.21530	3012	1	10	2019
Taboose Pass (TP)	36.96824	−118.43419	3321	3	18	2009
Big Pine Creek (BP)	37.12863	−118.48704	3142	11	28	1998–2014
Baker Creek (BK)	37.16780	−118.47143	3120	3	18	1999
S Bishop Creek (BC)	37.16601	−118.55171	3098	14	38	2004–2014
Tyee Lakes (TL)	37.18567	−118.57565	3191	4	9	2014
N Bishop Creek (NF)	37.21760	−118.64757	3131	6	12	2003–2014
Pine Creek (PC)	37.34442	−118.72861	3057	2	4	2013
Rock Creek (RC)	37.45561	−118.74034	3030	5	10	2013–2014
*Central Basin*						
Davis Creek (DC)	37.78392	−118.23650	2895	1	12	2003
*Eastern Cascades*						
Fitzhugh Creek (FC)	41.35091	−120.29662	1968	3	11	2020
*Coast Range*						
Gualala River (GR)	38.74906	−123.51919	12	1	8	2016

^a^We sampled newly emerged overwintered adults, either from the most recent population expansion (2013–2014), or the most recent observation of overwintered beetles at that site. Further details of sampling design are described in electronic supplementary material, appendix 1.1.

### (b) Microclimate simulation

The NicheMapR microclimate model simulated 24 variables for the 12 beetle populations that represent air, soil and snow conditions beetles experience throughout their life cycle (electronic supplementary material, table S3). Simulated environmental variables demonstrated high sensitivity to the shade input parameter in the model (electronic supplementary material, figure S1), but relative multivariate environmental distances between populations were consistent between minimum and maximum shade conditions (electronic supplementary material, figure S2). Simulated microclimatic data under minimum shade conditions were more concordant with available empirical measurement based on RMSE (electronic supplementary material, table S4), so downstream analyses were therefore conducted using simulated environmental variables under 10% shade.

### (c) Population genomic differentiation across California landscape

**Figure 1.**
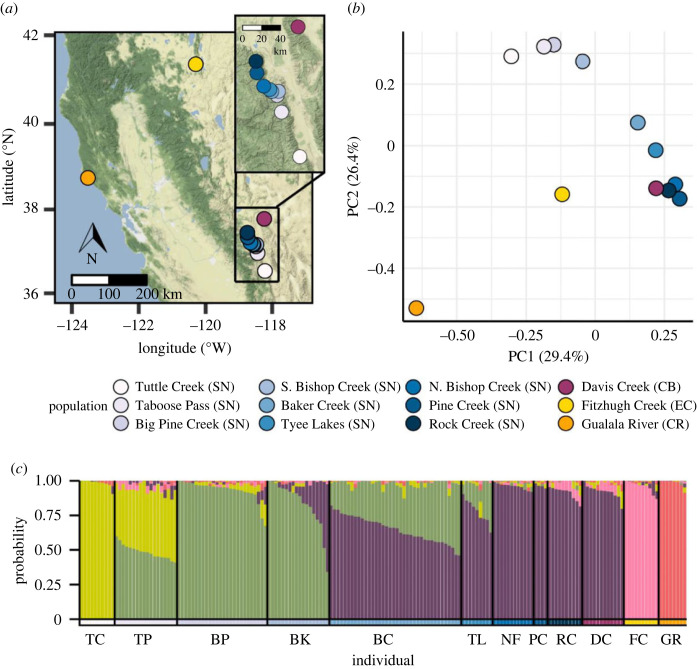
Genetic differentiation and structure of *Chrysomela aeneicollis* populations across California. (*a*) Map of study populations. Abbreviation in parentheses refers to population ecoregion (SN, Sierra Nevada; CB, Central Basin; EC, Eastern Cascades; CR, Coast Range). Inset map features the sampled populations located in the Sierra Nevada and Central Basin ecoregions. Populations in the Sierra Nevada ecoregion are presented using a blue colour gradient and are ordered based by latitude, south to north representing increasing latitude. (*b*) PCA ordination highlighting genomic differentiation among populations based on the minor allele frequencies. (*c*) Stacked barplots for each individual (*N* = 175 total) indicate estimated ancestry coefficients, representing the posterior probability that an individual originates from *K* = 5 ancestral gene pools. Colours below the stacked barplot indicate each individual's *a priori* population designations, as shown in (*a*,*b*). Two-letter population designations are described in table 1.

The first two principal components on population-level minor allele frequencies explained 55.8% of total genomic variation (figure 1*b*). Eastern Cascades and Coast Range (CR) ecoregions exhibited the greatest genomic divergence among populations, and population genomic variation in the SN and Central Basin ecoregions followed a latitudinal gradient (figure 1; electronic supplementary material, table S5). SNP filtering thresholds used in analyses did not meaningfully influence estimates of population structure compared to more relaxed filter thresholds (electronic supplementary material, figures S3–S5).

A population genetic structure analysis was used to estimate proportions of individual genomes originating from ancestral gene pools based on the five populations determined by selecting a value of *K* that minimized cross entropy (electronic supplementary material, figure S6). Individuals in the SN ecoregion show a strong pattern of genetic differentiation with latitude (figure 1). Based on proportions of estimated ancestral coefficients, individuals in the southern drainage Tuttle Creek (TC) are genetically distinct and belong to one ancestral population. Individuals in Taboose Pass (TP) are mixed, sharing ancestry with neighbours in TC to the south, Big Pine (BP) and Baker (BK) Creek to the north. Individuals in South Bishop Creek (BC) and Tyee Lakes (TL) share ancestry with both southern (BP, BK) and northern (NF, PC, RC) populations, which in turn share ancestry with those from the Great Basin (DC). Individuals collected in Eastern Cascades and CR ecoregions were genetically distinct from each other and from the SN–Great Basin complex (figure 1).

**Figure 2.**
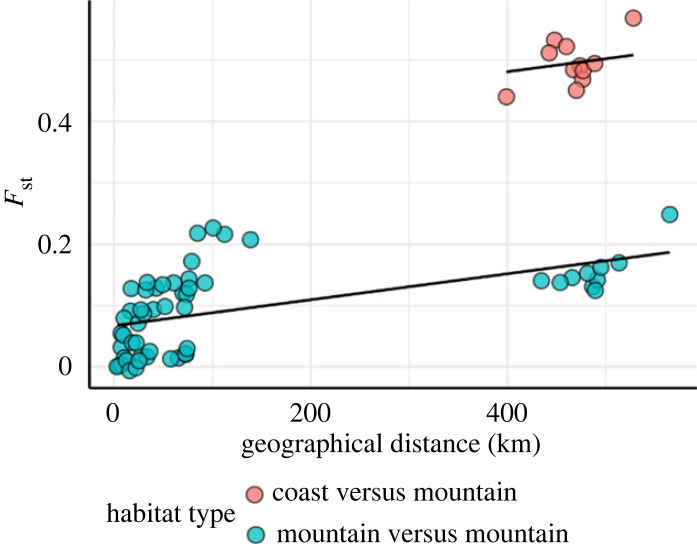
Genomic differentiation as a function of geographical distance and habitat type for California willow beetle populations*.* Data shown highlight the relationship between pairwise geographical distance (kilometres) and pairwise genetic distance (*F*_st_). The black lines indicate the fitted values from the ANCOVA model, and points are colour coded by the categorical independent variable used in the ANCOVA model.

Analysis of pairwise *F*_st_ values among population pairs revealed that populations in the montane Eastern Cascades region were more similar to montane populations in the SN and Great Basin than they were to CR populations, despite similar geographical distances separating each region (figure 2). When populations were classified by habitat type (coastal or mountain), *F*_st_ values for ‘coast versus mountain' population pairs were fourfold greater (LSM = 0.43 ± 0.02) than those for ‘mountain versus mountain' population pairs (LSM = 0.11 ± 0.01, *F*_1,63_ = 197.4, *p* < 0.001; figure 2). The overall relationship between geographical distance and *F*_st_ was similar within the two types of population pairs and was consistent with ‘IBD' genetic differentiation (*F*_1,63_ = 24.5, *p* < 0.001; figure 2). Together, these results suggest that IBD and isolation by environment (IBE) (coastal versus montane) both shape genomic differentiation, and differences in environmental conditions appear to strongly influence genetic composition of *C. aeneicollis* populations.

### (d) Associations between environmental and genomic variation

**Figure 3.**
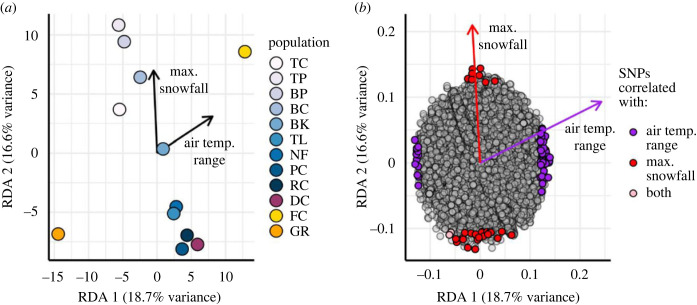
Partial redundancy analysis (pRDA) identifies candidate loci associated with selective climatic gradients. (*a*) Ordination of populations and environmental variable loadings in multivariate space. Environmental variable loadings are multiplied by 10 to improve visualization. (*b*) Ordination of SNP loci and environmental variable loadings in multivariate space. Outlier loci are coloured based on correlation with an environmental variable (Pearson's *r* > |0.65|). Environmental variable loadings are multiplied by 0.4 to improve visualization. Two-letter population designations are described in table 1. Results of pRDA with only montane populations are provided in electronic supplementary material, figure S7.

*Partial redundancy analysis* (*pRDA*)—The pRDA made it possible to identify specific genetic polymorphisms that were associated with environmental differences among populations. Among all California populations, the pRDA was globally significant (*F*_2,7_ = 2.18, *p* = 0.001; figure 3), and the constraining environmental matrix explained 17.1% of variation in genomic data, while the conditioning spatial matrix explained 19.0% of genomic variation. The forward selection procedure identified a significant positive spatial variable (MEM1), which was retained as conditioning variable in pRDA. The forward selection procedure identified annual air temperature range at 1.75 m above ground level (annual *T*_max_ − *T*_min_) and maximum daily snowfall as significant environmental predictors of genomic variation (figure 3; annual air temperature range *F*_1,7_ = 2.28, *p* = 0.001; maximum daily snowfall *F*_1,7_ = 2.08, *p* = 0.001). The first and second RDA axes also explained substantial proportions of genomic variation (RDA1 = 18.7%, *F*_1,7_ = 2.32, *p* = 0.014; RDA2 = 16.6% *F*_1,7_ = 2.05, *p* = 0.022). Candidate SNPs were identified based on high correlation with temperature- and snow-related environmental variables (*r* > |0.65|) and *z*-score values of loadings of loci in ordination space (*z*-scores ± 2.1, two-tailed *p* = 0.036). Based on these criteria, 107 SNPs were identified as candidate loci (figure 3). Sixty-eight SNPs were related to annual air temperature range, 37 to maximum daily snowfall, and two were related to both temperature and snowfall (electronic supplementary material, table S6). When the coastal Gualala River population was excluded, the pRDA was globally significant (*F*_1,8_ = 2.04, *p* = 0.021; electronic supplementary material, figure S7), and with MEM1 as the conditioning variable, the forward selection procedure identified only maximum daily snowfall as a significant predictor of genomic variation. Using the above candidate loci criteria, 116 SNPs were related to maximum snowfall (electronic supplementary material, table S7).

**Table 2.** Candidate proteins that vary with microclimate. Proteins were identified using BlastP alignment using predicted amino acid sequence; associated NCBI accession number is noted for sequence with highest homology to reference taxa; populations included in analysis (montane and coastal, montane only or both) are indicated.

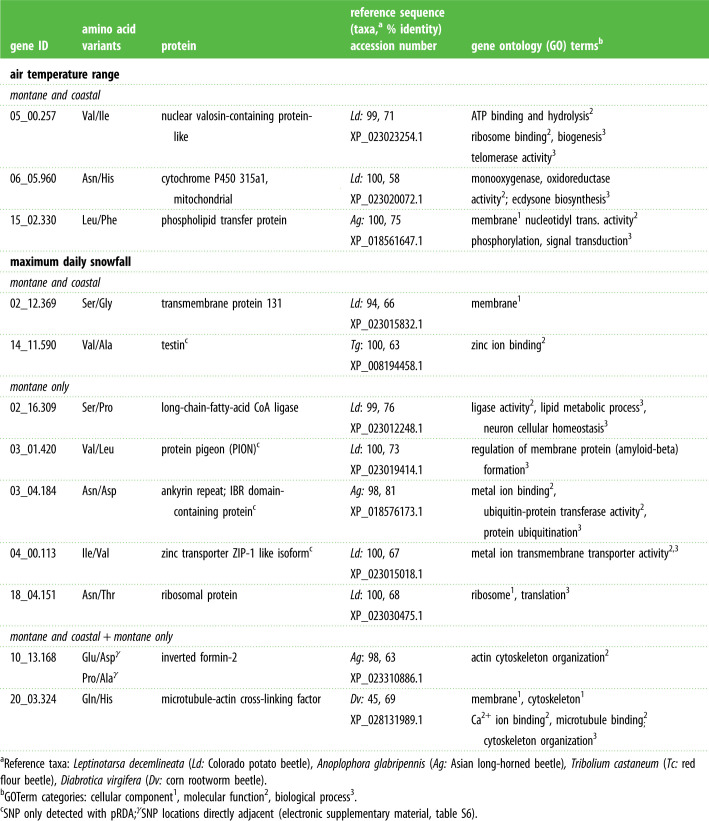

**Figure 4.**
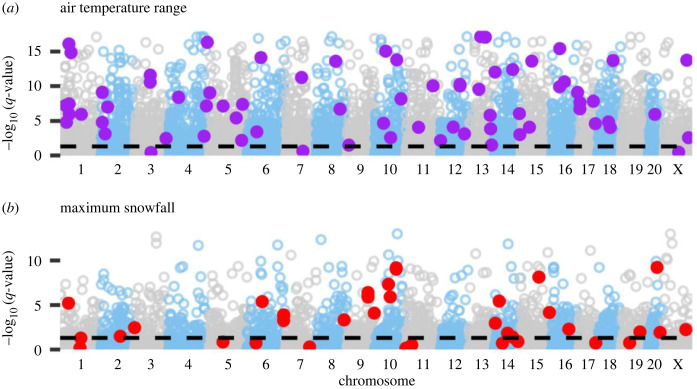
Latent factor mixed model (LFMM) identifies candidate loci associated with selective climatic gradients. Points indicate the FDR-adjusted *p*-value (*q*-value) of the association between a locus and an environmental gradient. The dotted black line represents a *q*-value of 0.05, and purple and red coloured loci are those detected by the pRDA. (*a*) Loci associations with annual air temperature range. (*b*) Loci associations with maximum daily snowfall. Results of LFMM with only montane populations are provided in electronic supplementary material, figure S8.

*Latent factor mixed model* (*LFMM*)—The LFMM represents a second approach to identify SNPs related to environmental variability while accounting for overall population genetic structure. With all California populations, a LFMM was run with five estimated ancestry coefficients as latent factors to test single-locus relationships with annual air temperature range and maximum daily snowfall (ancestry coefficients shown in figure 1). A large proportion of identified polymorphisms (19.2%; 4289 SNPs) were associated with annual air temperature range, and 7.2% (1603 SNPs) were associated with maximum daily snowfall (figure 4). Using the LFMM excluding the coastal Gualala River population and four ancestral populations, 1471 SNPs (6.6% total) were associated with maximum snowfall (electronic supplementary material, table S7 and figure S8).

### (e) SNP and protein functional annotations

To reduce probability of false positive associations and narrow the search for candidate polymorphisms, we focused on SNPs identified by both pRDA and LFMM, and we assessed associations across coastal and montane populations and then again using only montane populations (electronic supplementary material, tables S6 and S7). In analyses with all populations, most SNPs correlated with annual air temperature range in pRDA were also identified using LFMM (67 of 70). A slightly lower proportion of SNPs associated with maximum daily snowfall based on pRDA were also identified by LFMM (26 of 39; electronic supplementary material, table S6). In analyses with only montane populations, most SNPs correlated with maximum daily snowfall based on pRDA were also identified by LFMM (79 of 116), and 18 SNPs identified in the pRDA with only montane populations were also identified in analyses with coastal and montane populations (electronic supplementary material, table S9).

Analyses of all populations and only montane populations identified three non-synonymous SNPs associated with maximum daily snowfall found in genes coding for proteins involved in cell structure and movement (inverted formin-2 and microtubule-actin cross-linking factor). Analyses including all populations or only montane populations identified five non-synonymous SNPs associated with daily snowfall that are found in genes coding for proteins involved in ion transport or cellular membrane activity (table 2; electronic supplementary material, tables S8–S12). Three non-synonymous SNPs associated with air temperature range across all populations were found in genes coding for proteins involved in intracellular signalling and energetics (cytochrome p450, phospholipid transfer protein; table 2).

## 3. Discussion

Detecting accurate signals of local adaptation in the genome requires linking observed genetic patterns to underlying selective features of the environment while accounting for associations imposed by neutral processes. Here, we demonstrate that populations of the willow leaf beetle *C. aeneicollis* across California are differentiated across the nuclear genome, and we provide strong evidence that snow serves as a prominent selective gradient and driver of local adaptation across their geographical range. We show that both large-scale variation in snowfall across the California landscape and small-scale variation in snowfall within montane locations are associated with adaptive genetic variation. Specifically, we provide evidence that variation in maximum daily snowfall is linked to non-synonymous polymorphisms in genes associated with cytoskeletal motility, ion transport, and membrane structure and function, highlighting the potential role of adaptive protein modifications that could enhance insect cold tolerance in cold snowy regions.

### (a) Spatial patterns of genetic divergence in *Chrysomela aeneicollis*

Results of this study reveal that populations of the willow leaf beetle *C. aeneicollis* living in different regions of California are genetically differentiated across loci in the nuclear genome. Three of four ecoregions sampled show substantial levels of genetic divergence among them, such that North Coast populations are distinct from those in the Eastern Cascades ecoregion, and both of those populations are distinct from populations in the SN and Great Basin (figure 1). Within eastern California, populations from northern drainages of the eastern SN are less genetically isolated from populations sampled in the neighbouring White Mountains than those found in southern drainages of the SN. Furthermore, consistent with previous studies, populations within the SN show relatively high levels of genetic divergence given their relatively close geographical proximity (figure 1) [3,42,43]. Patterns of genetic differentiation separating populations in different ecoregions suggest that geographical and seasonal environmental variation present a major selective pressure on alleles in the nuclear genome and that genes related to thriving under different local environmental conditions contribute to local adaptation among populations of *C. aeneicollis* (figure 2).

The strong pattern of geographical differentiation of genomic variation across California was illustrated for the first time in the present study, but it is consistent with findings of Dellicour *et al*. [40]. The earlier study found that *C. aeneicollis* populations in western North America (Montana, coastal Oregon, Colorado and California) were strongly differentiated at mitochondrial and nuclear genetic markers, suggesting that geographical isolation among these regions predates recent fluctuations in the extent of glaciation over the past 50 000 years. Isolation of populations at mitochondrial loci was greater than nuclear genes, but there was overall agreement among loci that differentiation among geographical regions was substantial, which would contribute to conditions favouring local adaptation [40]. To date, this study provides the best picture of signatures of adaptation to seasonal variation in this wide-ranging insect.

### (b) Maximum daily snowfall variation contributes to adaptive genetic variation

Identifying climatic variables that act as drivers of spatially varying selection will be critical for predicting evolutionary responses to climate change and environmental disturbance [48]. Among all simulated microclimate conditions that represent air, soil and snow conditions throughout the year, we found that maximum daily snowfall explains a significant portion of variation in genomic data, after controlling for spatial autocorrelation and population history (figure 3; electronic supplementary material, figure S7). This association is identified both across the California landscape, where climatic conditions differ greatly between coastal and montane populations, as well as within montane populations, where differences in climatic conditions are more subtle. Comparisons made within montane populations suggest that this snowfall gradient may characterize spatially varying selective pressures related to winter cold exposure within mountain ecoregions (electronic supplementary material, figure S9). Eco-physiological models for *C. aeneicollis* indicate that the relationship between elevation and cold exposure in soil is strongly nonlinear, with cold exposure peaking at mid-elevation montane populations that are not buffered by persistent snow cover [38]. Since snow decouples the relationship between air and soil temperatures, variation in snow cover reflects variation in cold exposure in the soil at a given elevation. Without the thermal buffer that snow provides for organisms overwintering in soil, cold microclimate temperatures can drop below a species-specific cold tolerance threshold, which can result in mortality or sublethal cold injuries [49,50]. This result highlights the importance of snow cover variation as a key factor in maintaining this variation and driving selection on genes associated with cold tolerance and stress in winter.

Prior work in SN populations of *C. aeneicollis* shows that air temperature varies between genetically differentiated populations and shows evidence of physiological adaptation to different thermal regimes [44]. However, in the present study, the effect of ‘annual air temperature range' (figure 3) is largely driven by climatic conditions in the CR. This broad thermal selective gradient covaries with neutral patterns of population structure, which complicates distinctions between neutral and selected loci [24]; thus, the high detection rates observed in the LFMM in the present study could also be due to residual, unaccounted population structure (figure 4).

### (c) Putative mechanisms of local adaptation to snow cover mirror mechanisms of cold tolerance

Genes containing non-synonymous SNPs associated with variation in snowfall encode proteins with functions related to ion binding, actin and cytoskeleton binding and organization, and membrane components; protein identifications were assigned with a high level of confidence, as all homologous proteins are present in other beetle species (table 2). These protein functions align with previously identified mechanisms of cold tolerance and acclimation in both insects and plants [51–54]. Primary cellular challenges associated with deep and prolonged cold exposure or freezing include loss of ion and water homeostasis and depolymerization of cytoskeletal components (e.g. actin and tubulin), which can impair ion transport function, cause loss of cell junction integrity, and exacerbate disturbances in membrane integrity caused by paracellular leaks of water and ions [55–58]. Cold-acclimated insects are better able to maintain ion and water balance at low temperatures compared to warm-acclimated insects [59], due to cellular structural modifications that enhance cytoskeletal stability, thus protecting ionoregulatory tissues (e.g. Malpighian tubules in insects) from chilling injury and loss of transport function [51]. Cold-acclimated insects also differentially regulate cytoskeletal gene expression, with cold acclimation inducing upregulation of actin-associated genes or enzymes that promote membrane and cytoskeletal remodelling [52,60,61]. Because polymorphisms associated with variation in snowfall may relate to protein modifications that enhance cytoskeletal and membrane stability in the cold, putative mechanisms underlying local adaptation to snow are related to primary cellular mechanisms of cold acclimation and tolerance. These results provide genomic evidence that variation in snowfall imposes a selective gradient in exposure to cold stress, supporting the theory that snow modulates cold stress and exposure for insects that overwinter in the soil [38].

### (d) Tandem genotype–environment association approach identifies signatures of local adaptation

In detecting genomic signatures of local adaptation, genotype–environment associations (GEAs) identified by various methods will depend strongly on demographic and sampling scenarios [24,29,31,62]. Simulations conducted by [23] find that multivariate ordination methods like pRDA produce uniformly low false positive rates (0–2%), whereas LFMM produced high false positive rates under low dispersal scenarios [23]. *Chrysomela aeneicollis* individuals have low levels of dispersal, with individuals often spending most of their life on a single host plant [41,63].

Nonetheless, correcting for population structure in pRDA can result in low power to detect true associations [64], and recent simulation modelling indicates that LFMM provides the best compromise between detection power and error rates in situations with complex hierarchical neutral genetic structure [65]. Herbivorous insects can have a subdivided population structure that reflects the distribution of their plant hosts [66,67], and previous work found hierarchical, subdivided genetic structure among patches and willows within a patch [43]. The application of these two GEA methods highlights the trade-off between conservative and liberal approaches in detecting a true adaptive signal, yet applying these methods in combination can therefore yield increased confidence in true positive detections of local adaptation. Future work should investigate the relationship between non-clinal allele frequency patterns and environmental gradients, which can evolve under multivariate environments and can lead to inaccurate inferences using GEA approaches [64].

### (e) Limitations

A limitation of this study is sampling bias toward populations in the SN ecoregion relative to the other three eco-regions included in this study (figure 1), which may bias genetic–environmental relationships and relative contributions of IBE and distance (figure 2). Replicated sampling along environmental gradients increases confidence in true positive detections of GEAs [31], yet the beetle's fragmented distribution in California limits replication across climatic conditions. Another potential limitation is that temporal coverage of sampling was limited to 1 year in all but the SN ecoregions (table 1), so that allele frequencies in these populations may be influenced by environmental conditions in the collection year. Prior studies suggest that genetic variation among SN, CB and CR populations has remained relatively stable since we last sampled and analysed them [40]. We therefore expect that patterns reported here reflect adaptation to long-term environmental conditions due to the geographical isolation among populations.

Additionally, stringent SNP filter thresholds were applied to ensure quality genotypes within each population, resulting in a relatively modest set of polymorphisms (*N* = 22 323 SNPs). While these thresholds did not alter overall estimates of population structure (electronic supplementary material, figures S3–S5), candidate SNPs associated with environmental gradients in this study likely represent a subset of loci involved in local adaptation.

## 4. Conclusion

Many montane species live on the periphery of both suitable habitat and physiological tolerance, which contributes to the unique sensitivity of montane populations to climate change. Even small environmental changes may result in large implications for survival and reproductive success [33,68,69]. The willow leaf beetle has emerged as a natural model for analysing the relationship between adaptive genetic variation and environmental change [33,42–44]. By analysing all known Californian *C. aeneicollis* populations across the nuclear genome, this study represents the broadest investigation of adaptive genetic variation in the species to date and provides a path forward for understanding the evolutionary significance of variation at genes associated with response to environmental stress. Future work should identify regions where genetic–environmental relationships will be most likely disrupted by climate change and reduced snowfall, which will be critical for land management decisions and gene conservation in vulnerable populations [70].

## 5. Methods

### (a) Study populations and sampling design

Ecoregions were identified following United States Geological Survey (USGS) designations [47]. Beetle populations from the SN ecoregion were surveyed at winter snowmelt (May–June) from 1996 to 2016, following methods detailed in [33] (electronic supplementary material, appendix 1.1). In all, 175 individuals from 54 sampling locations were included and assigned *a priori* to 12 populations (table 1 and figure 1) based on previous work [40,42,43]. These represent all known populations in California, and they experience a wide range of seasonality, snow cover and air temperature variation, especially between montane and coastal regions (electronic supplementary material, table S3). Though allele frequencies can fluctuate within a beetle population across years [18], the magnitude of these fluctuations is relatively small compared to the magnitude of genetic divergence among regions [3,40,42].

### (b) DNA library preparation and processing of genomic sequencing data

Genomic DNA was extracted from individual beetles using NucleoMag Bacteria DNA Isolation kit (Macherey-Nagel, Düren, Germany), and whole-genome libraries were prepared following the plexWell library preparation protocol by the CCGP MiniCore. Paired-end sequencing (2 × 150 bp) was performed on an Illumina HiSeq4000 platform at UC Berkeley's QB3 Genomics Core Facility (Berkeley, CA, USA). Nextera adapter sequences and low-quality bases (base quality <15, sliding window 4 bp) were removed from each read using Trimmomatic v. 0.39 [71]. Reads were aligned to a *C. aeneicollis* reference genome [46] using the Burrows-Wheeler Aligner (BWA-MEM) algorithm [72]. Joint genotyping was performed on all samples using Genome Analysis Toolkit (GATK) v. 4.2.6.0 functions HaplotypeCaller and GenotypeGVCFs [73]. Variant data were filtered to include only biallelic SNPs, and SNPs were hard-filtered using GATK best-practice recommendations [74] (electronic supplementary material, table S2*a*). SNPs were removed if MAF across all individuals was less than 0.01 or if heterozygote frequencies deviated greatly from Hardy–Weinberg expectations (e.g. excess heterozygosity or inbreeding coefficient greater than ±0.5) (electronic supplementary material, table S2*b*). Finally, SNPs were retained if 70% of all samples and 70% of samples within each population showed a read depth between three and 30 and a genotype quality greater than 20 (electronic supplementary material, table S2*c*) [75]. After filtering, principal components analysis (PCA) was performed on Hellinger-transformed population-level minor allele frequencies [75,76]. Because variant filter thresholds influence estimates of population structure [77,78], we assessed sensitivity of genetic differentiation to filter threshold levels.

### (c) Microclimate variable simulation

To obtain spatially explicit environmental variables representing local microclimate conditions across the life cycle, microclimate simulations were conducted for the 12 beetle populations using the biophysical modelling package *NicheMapR* [79]. The model computes microclimatic conditions at a defined distance above ground, given local habitat properties and weather conditions. The microclimate model was run using historical gridded weather data from the GRIDMET daily weather database with 5 km × 5 km resolution [80]. The mid-latitude, -longitude and -elevation of all demes within each population were used as input in the model (table 1; electronic supplementary material, table S2). The microclimate model was run in soil moisture and snow modes under both minimum (10%) and maximum shade (90%) conditions for 1989–2020. Simulated variables included air temperature and humidity at 1.75 m above the ground, snow-related variables and soil-related variables. To characterize mean environmental conditions, daily microclimate variables were averaged over 30 simulated years (electronic supplementary material, table S3). We evaluated sensitivity of simulated microclimate variables to input microclimate model parameters by calculating RMSE between simulated outputs and empirically derived microclimate data from available weather stations (California Department of Water Resources, CDEC). Air temperature and snow depth data from CDEC were available for weather stations within 1 km of mid-elevation sites in Rock Creek, BP Creek, South Bishop Creek and North Bishop Creek.

### (d) Population genomic differentiation across the California landscape

Population structure from SNP genotypic data was assessed by estimating proportions of individual genomes originating from ancestral gene pools. A range of estimated ancestral gene pools (*K* = 1–10) were tested using a sparse non-negative matrix factorization algorithm using the function ‘snmf' in the R package *LEA* v. 3.6.0 [81,82] (*K* = 3–7 shown in electronic supplementary material, figure S11). The value of *K* that minimized cross-entropy and best explained genotypic data was five [83] (electronic supplementary material, figure S6) and this value was used for subsequent analysis. The ‘snmf' function was also used to estimate individual ancestry coefficients. Five replicates were run using the best estimate of *K*, and individual ancestry coefficients were extracted from the replicate with the lowest cross-entropy.

To quantify contributions of geographical and environmental distances to patterns of genetic differentiation, we assessed IBD and IBE for all population pairs using an analysis of covariance (ANCOVA). The ANCOVA tested whether the means of pairwise *F*_st_ between populations were equal across habitat type, while controlling geographical distance. Unbiased pairwise *F*_st_ using minor allele frequencies of all populations were calculated using the R package *BEDASSLE* v. 1.6 [84,85]. Pairwise geographical distance in kilometres was calculated using the R package *fields* v. 13.3 [86]. Population pairs were identified as ‘coast versus mountain' and ‘mountain versus mountain' to describe habitat type of populations, as this categorical descriptor represents most environmental variation among populations (electronic supplementary material, table S3). Using the R package rstatix v. 0.7.1, ANCOVA was conducted with pair-wise *F*_st_ values as dependent variable, binary environmental descriptor as categorical independent variable, and geographical distance as a covariate. Least-squares means were calculated for habitat types using the R package *emmeans* v. 1.8.3.

### (e) Genotype–environment association tests to identify signatures of local adaptation

Signatures of local adaptation to climate were investigated using two GEA methods, pRDA [29,87,88] and LFMM [30], which control for signals generated by neutral processes through separate mechanisms. Both GEA analyses were performed on two sets of populations: (i) all populations and (ii) all montane populations excluding the coastal (Gualala River) population. pRDA was conducted at the population level since the resolution of environmental data did not include environmental variation within a population. To account for IBD, we conducted a spatial eigenfunction analysis that produced a conditioning matrix in the pRDA using distance-based Moran's eigenvector maps (electronic supplementary material, appendix 1.2) [29]. All simulated environmental variables were scaled and centred to produce the environmental matrix, and forward selection was used to select significant environmental predictors, with significant dbMEMs as explanatory conditioning matrix and Hellinger-transformed SNP minor allele frequencies as response matrix. The final pRDA was run with significant (alpha < 0.05) environmental predictors using the R package *vegan* v. 2.6-2 [89]. Outlier loci on constrained ordination axes were determined based on loadings of each locus in ordination space [29,75].

We then conducted a LFMM and documented overlap of detections with results from pRDA [24,29,48,65]. Neutral population structure due to shared demographic history or background genetic variation is introduced through unobserved, latent factors [30]. This method used individual-based genotypic data, which assessed the effect of *a priori* designated populations used previously in pRDA. The ‘lfmm' function in the *LEA* package was implemented using individual-level genotypic data (22 323 SNPs) as response matrix, forward-selected environmental variables used in pRDA as environmental predictors, and the best estimate of *K* (estimated ancestral gene pools) as number of latent factors. More detailed GEA methods are provided in electronic supplementary material, appendix 1.3.

### (f) SNP and protein functional annotations

We identified genes containing candidate SNPs and predicted SNP coding effects with an interval forest approach using the program SnpEff v. 5.1 [90] and Caen 1.0 annotated genome [46]. SNPs were annotated based on genomic location, and coding effects were predicted (electronic supplementary material, appendix 1.4). To assign a putative protein name, protein sequences were aligned to NCBI's protein database using BlastP (table 2; electronic supplementary material, tables S8–S11). Gene ontology (GO) terms were assigned to candidate genes using the functional annotation Web server database Protein ANNotation with *Z*-scoRE (PANNZER2 [91]).

## Data Availability

All code and analyses are published in Figshare: https://doi.org/10.6084/m9.figshare.22272025.v4 [95]. Supplementary materials are provided online [96].
